# Screening of Antibacterial Producing Actinomycetes from Sediments of the Caspian Sea

**Published:** 2013

**Authors:** Mojtaba Mohseni, Hamed Norouzi, Javad Hamedi, Aboulghasem Roohi

**Affiliations:** 1 *Department of Molecular and Cell Biology, University of Mazandaran, Babolsar, Iran.*; 2 *Department of Microbiology, University of Tehran, Tehran, Iran.*; 3 *Caspian Sea Ecology Research Institute, Sari, Iran.*

**Keywords:** Marine actinomycetes, antimicrobial activity, caspian sea

## Abstract

Actinomycetes are interesting as a main producer of secondary metabolites and industrial antibiotics from marine environments. A total of 44 strains of actinomycetes were isolated from Caspian Sea sediments at a depth of 5-10 m. Preliminary screening was done using cross-streak method against 2 gram-positive and 4 gram-negative pathogen bacteria. The most potent strains MN2, MN3, MN38, MN39, MN40, MN41, and MN44 were used to extract the antibacterial substances. The antibacterial activities were performed using Kirby-Bauer disk diffusion method. Potent actinomycetes were screened for hydrolytic exoenzymatic activities (amylase and protease). All of the 24 isolates were active against at least to one of the test organisms. The MN38 strain showed activity against *Staphylococcus aureus* (20.0±0.5mm), *Bacillus subtilis* (27.0±0.2 mm), *and Escherichia coli* (20.0±0.3 mm). The MN39 strain was also active against *E. coli* (23.0±0.4mm), *B. subtilis* (23.0±0.2mm), *Klebsiella pneumonia* (24±0.1mm), whereas, the MN3 strain showed activity against *Pseudomonas aeruginosa* (20.0±0.2mm). The results of this investigation revealed that the marine actinomycetes of Caspian Sea sediments were potent source of novel antibiotics and bioactive compounds.

Actinomycetes are free living, saprophytic, filamentous bacteria, and a major source for the production of antibiotics (1). They are found in soil, fresh water and marine water environments (2). Actinomycetes provided many important bioactive compounds of high commercial value and screened for new bioactive substances (3). These bacteria are an important group of microorganisms due to their ability to produce a wide array of secondary metabolites, such as antibiotics, antitumor agents, immunosuppressive agents, cosmetics, vitamins, nutritional materials, herbicides, pesticides, anti-parasitic agents and enzymes (1, 4, 5). Around 23000 bioactive secondary metabolites produced by microorganisms have been reported. Over 10000 of these compounds are produced by actinomycetes, representing 45% of all bioactive microbial metabolites discovered. Among actinomycetes, around 7600 compounds are produced by *Streptomyces* species (6). As the frequency of novel bioactive compounds obtained from terrestrial actinomycetes decreased, it had been emphasized that actinomycetes from marine sediments might be valuable for the isolation of novel strains which could potentially yield a broad spectrum of secondary metabolites (7-9). However, it has been resolved whether actinomycetes forms part of the marine microbial community of sediment samples originated from terrestrial environments and was simply carried out to sea in the form of resistant spore (10). 

It has been reported that marine actino-mycetes not only have several new species, but also have plenty novel structures with potent bioactivities (11). Several novel bioactive compounds were discovered from aquatic actinomycetes for example rifamycin from *Micromonospora *sp. (12); salinosporamide-A, an anticancer metabolite from *Salinispora* sp. (13) (Feling, 2003); marinomycins from *Marinophilus* sp. (14); abyssomicin-C from *Verrucosispora* sp. and marinopyrroles from *Streptomyces* sp. (12, 15). The appearances of multidrug resistant pathogenic strains caused substantial morbidity and mortality especially among the elderly and immunocompro-mised patients. To overcome this situation, there is an interest to improve or discover novel class antibiotics that have different mechanisms of action worldwide (16). 

According to incomplete statistics, the number of novel compounds obtained from marine actinomycetes in the 21^st^ is more than twice of the last century. This study was focused on the actinomycetes of marine sediments collected from the Caspian Sea. For the first time, an effort was made to screen different marine sediments which are a large unscreened and diverse ecosystem for the isolation of potent antibiotic producing actino-mycetes.

## Materials and Methods

**Sample collection**

Samples were collected from the sediments of Caspian Sea at the depths of 5-10 m by Van vein grab (0.2 m^2^). Two sampling stations were located along of the Caspian Sea with the following latitudes 36°43'N and 36°44'N. The surface of each grab sample was aseptically collected and processed within 30 minutes.

**Sample treatment**

The samples were subjected to physical pretreatment method in order to facilitate the isolation of actinomycetes. Heat treatments were performed by holding sediment samples in a water bath (Memmert) at 50 °C for 60 minutes. All samples were diluted with sterile 0.9 % saline prior to inoculation in triplicate onto isolation plates (11).

**Isolation of** **actinomycetes**

Actinomycetes were isolated by serial dilution method from sediments (17). Stock solution was prepared by diluting 1 g of sediment in 9 ml of sterile saline water and shaking well by using a vortex mixer (IKA). From the stock solution, 1 ml was used to prepare the final volume of 10^-2^ and 10^3^ by serial dilution method. Samples were inoculated on Starch Casein Agar (SCA) (composition: soluble starch: 10 g, K_2_HPO_4_: 2 g, KNO_3_: 2 g, casein: 0.3 g, MgSO_4_.7H_2_O: 0.05 g, CaCO_3_: 0.02 g, FeSO_4_.7H_2_O: 0.01 g, agar: 15 g, filtered sea water: 1000 ml and pH: 7.0±0.1), Yeast Extract Malt Extract Agar (ISP2) (Composition: yeast extract: 4 g, malt extract: 10 g, dextrose: 4 g, agar: 15 g, filtered sea water: 1000 ml and pH: 7.3) and Kuster's Agar (composition: glycerol: 10 g, casein: 0.3 g, KNO_3_: 2 g, K_2_HPO_4_: 2 g, soluble starch: 0.5 g, asparagine: 0.1 g, FeSO_4_.7H_2_O: 0.01 g, CaCO_3_: 0.02 g, MgSO_4_.7H_2_O: 0.05 g, agar: 15 g, filtered sea water: 1000 ml and pH: 7.0±0.1). Each medium was supplemented with 25 µg ml^−1^ nystatin to minimize contamination with fungi and 10 μg ml^−1^ nalidixic acid to minimize contaminant growth (11, 18). Plates were incubated for 7 to 20 days at 28 °C. Then the colonies with a tough or powdery texture, dry or folded appearance and branching filaments with or without aerial mycelia were sub-cultured on slants SCA (19). Until further use, the slants were kept in cold room at 4 °C (6).

**Preliminary screening for antibacterial activity using cross-streak method**

Isolated strains MN1 to MN44 were inoculated onto nutrient agar plates by streak in the center. The plates were incubated at 28 °C for 3 days. Six bacteria including *Staphylococcus aureus *ATCC 25923, *Bacillus subtilis *PTCC 1156, *Escherichia coli *PTCC 1533, *Pseudomonas* *aeruginosa* PTCC 1074, *Salmonella* *typhi* PTCC 1609 and *Klebsiella* *pneumonia* were used as test organisms. A pure colony of test bacteria was transferred into fresh nutrient broth and incubated at 37 °C for 24 hours until the visible turbidity and density equal to that of 0.5 McFarland. After adjusting the turbidity, sterile cotton swab was dipped into the bacterial suspension and streaked perpendicular to the antagonist on the agar medium. The plates were incubated at 37 °C for 24 hours. The microbial inhibitions were observed by determining the diameter of the inhibition zones. 

**Extraction of antimicrobial compounds**

The selected antagonistic actinomycetes were inoculated into 100 ml of actinomycete isolation broth (composition: glycerol: 5.0 g, sodium propionate: 4.0 g, sodium caseinate: 2.0 g, K_2_HPO_4_: 0.5 g, asparagine: 0.1 g, MgSO_4_·7H_2_O: 0.1 g, FeSO_4_·7H_2_O: 1.0 mg, water: 1000 ml and pH: 8.0±0.1) and incubated in orbital shaker at 28 °C and 190 rpm for 7 days. To extract the antimicrobial compounds, the cultures were filtered then centrifuged (Sigma) at 6000 rpm, 10 minutes (1). The supernatant was transferred aseptically into a screw capped bottle and stored at 4 °C for further assay.

**Secondary screening of antibacterial activity using disk diffusion method**

The antimicrobial activities of those extracts were tested against different test organisms by using agar disc diffusion method as described by Kirby-Bauer with modification (20). 

Late exponential phase of the test bacteria were prepared by inoculating 1% (v/v) of the cultures into the fresh Muller-Hinton broth (Merck) and incubating on an orbital shaker at 37 °C and 100 rpm overnight. Before using the cultures, they were standardized with a final cell density of approximately 10^8^ cfu ml^-1^. Muller-Hinton agar (Merck) were prepared and inoculated from the standardized cultures of the test organisms then spread as uniformly as possible throughout the entire media. Sterile paper discs (6 mm diameter, Padtan, Iran) were impregnated with 30 µl of the extracts then allowed to dry. The impregnated disc was introduced on the upper layer of the seeded agar plate and incubated at 37 °C for 24 hours. 

The antibacterial activities of the extracts were compared with known antibiotic tetracycline (30 µg/disc) as positive control and ethyl acetate (30 µl/disc) as negative control. Antibacterial activity was evaluated by measuring the diameter of inhibition zone (mm) on the surface of plates and the results were reported as Mean ± SD after three repeats (21). The potential actinomycetes isolates were selected from the primary and secondary screening then characterized by morphological and physiological methods for further studies. 

**Exoenzymatic assay**

The potential actinomycetes isolates were screened for hydrolytic exoenzymatic activities including amylase and protease. These tests were conducted on Yeast Extract Malt Extract Agar (ISP2) containing 1% soluble starch for amylolytic activity and 1% skimmed milk for proteolytic activity.The presence of amylase was visualized by decolorized halo around the culture due to starch digestion. Proteolytic activity was observed by clearing of the milk around the colony (6).

## Results

**Fig 1:**
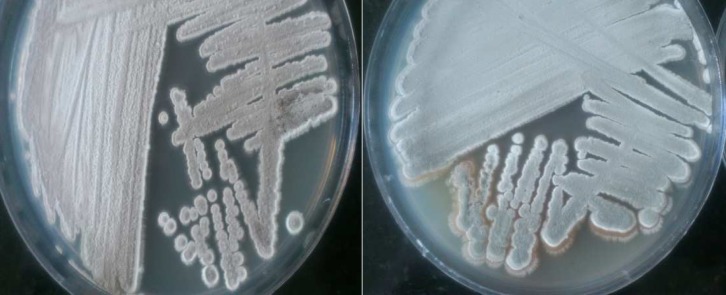
Morphological appearance of MN38 isolates (A) and MN39 (B) on Starch Casein Agar

**Fig 2:**
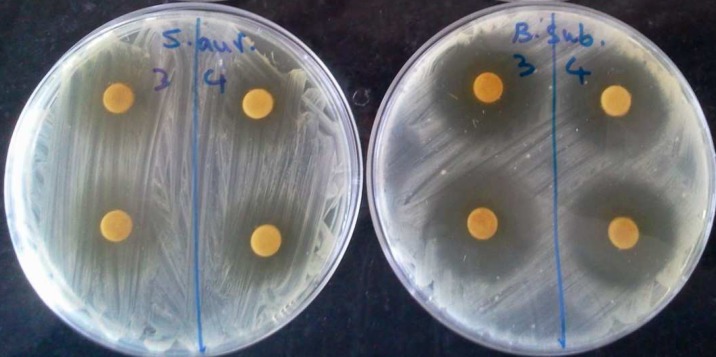
Antibacterial activities of the crude extract of MN38 isolates (3) and MN44 (4) against S. aureus (A) and B. subtilis(B) using disc diffusion method

Actinomycete colonies were readily isolated from marine sediments on SCA and ISP 2, but the growth on Kuster's agar was very poor. The macroscopic appearance of the isolates showed leathery and powdery colonies in Starch Casein Agar (SCA) (Fig. 1). A total of 44 actinomycetes were isolated from the sediments of Caspian Sea. Among the isolated actinomycetes, 24 strains showed antibacterial activities against at least one of the tested bacteria using preliminary screening. The results were summarized in table 1. Of all the 24 isolates, seven best antagonistic actinomycetes isolates were selected for further studies. The potential isolates are MN2, MN3, MN38, MN39, MN40, MN41, and MN44. The morphological characteristics of the selected isolates are shown in table 2. The strains were gram positive, filamentous with long spore chain except the MN39 which produced single spore. For antibacterial activities assay, the crude extracts of the potential isolates were subjected for secondary screening using disc diffusion methods (Fig. 2). The results demon-strated that among the tested bacteria, the crude extracts exhibited highest antibacterial activity against *B. subtilis* and *S. aureus* (Table. 3). The values obtained for activity of MN38 and MN39 strains show great activity against all test bacteria whereas the growth inhibitory of the crude extract of MN3 strain showed low activityagainst *K. pneumonia* (Table 3). The results revealed that MN44, MN38 andMN39 were active against *S. aureus*; MN41, MN38 against *E. coli*; MN2, MN3 and MN44 against *P. aeruginosa*; MN2, MN39, MN40 and MN44 against *K. pneumonia*; MN38, MN39 and MN44 against *B. subtilis*; MN40 and MN44 against *S. typhi*. Physiological and biochemical characteristics indicate that all isolates showed the ability of starch and protein hydrolysis.

**Table 1:** Preliminary screening of actinomycetes for antimicrobial activity using cross-streak method

Isolates	**Test bacteria**					
	*E. coli*	*B. subtilis*	*K. pneumonia*	*S. aureus*	*S. typhi*	*P. aeruginos*
MN1	-	+	-	+	-	+
MN2	+	+	+	+	+	+
MN3	+	+	+	+	+	+
MN6	-	+	-	+	-	+
MN8	+	-	-	+	-	+
MN13	-	+	-	+	-	+
MN16	-	+	-	+	-	+
MN17	+	+	-	+	-	-
MN18	-	+	-	+	-	+
MN19	-	+	-	+	-	+
MN21	-	+	-	+	-	+
MN23	-	+	-	+	-	+
MN27	-	+	-	+	-	+
MN30	+	+	-	-	-	+
MN31	-	+	-	+	-	+
MN32	-	+	-	+	-	-
MN33	+	+	-	+	-	+
MN35	-	+	-	+	-	+
MN36	-	+	-	+	-	+
MN38	+	+	+	+	+	+
MN39	+	+	+	+	+	+
MN40	+	+	+	+	+	+
MN41	+	+	+	+	+	+
MN44	+	+	+	+	+	+

**Table 2:** Morphological and physiological characteristics of potent actinomycetes isolates

**characteristics**	**Isolates**						
	MN2	MN3	MN38	MN39	MN40	MN41	MN44
Aerial mycelia	Green	Gray	Gray	White	Gray	White	White
Reverse color	Yellow-brown- orange	Yellow-brown-green	Yellow-brown- green	Yellow-brown- red	Yellow-brown	Yellow-brown	Yellow-brown
Spore-bearing hyphae	Verticillate (MV)	Simple (R)	Simple (F)	Simple (RA)	Simple (R)	Verticillate (BIV)	Simple (R)
Starch hydrolysis	+	+	+	+	+	+	+
Casein hydrolysis	+	+	+	+	+	+	+

MV):Monoverticillus, (R): Straight - Rectus, (F): Flexibilis, (RA): Retinaculum-Apertum, (BIV): Biverticillus-: Negative; +: Positive.

**Table 3:** Antibacterial activity of the potential actinomycetes isolates using Kirby–Bauer disk diffusion method

**Test Strain**	**Zone of growth inhibition (mm)**							
	MN2	MN3	MN38	MN39	MN40	MN44	MN41	Tet.
*E. coli*	13.0±1.4	17.0±0.7	11.0±1.4	23.5±0.7	14.0±1.4	13.0±1.1	20.7±1.5	18.6±1.1
*P. aeruginosa*	18.0±0.7	20.0±0.7	15.5±0.7	11.0±1.4	12.5±0.7	18.0±0.5	10.0±1.5	NE
*S. aureus*	10.5±0.7	13.0±1.4	20.0±0.4	20.5±0.7	12.5±0.7	20.3±1.5	12.0±0.4	24.0±1.0
*B. subtilis*	12.0±0.5	11 0±0.6	27.0 ±0.7	23.0±1.4	13.0±0.7	22.0±1.7	13.0±1.4	23.6±0.6
*S. typhi*	14.0±1.4	14.0±1.4	16.0±1.4	16.0±1.4	18.0±1.4	20.0±1.4	11.0±1.7	19.3±0.6
*K. pneumonia*	17.0±0.4	7.0±1.4	15.0±1.4	24.0±1.4	17.0±1.4	18.0±1.4	10.0±1.4	14.3±0.6

Tet.: Tetracycline (30µg/disk)           NE: No Effect

## Discussion

Currently, the incidence of multidrug resistant organisms is increasing and compromising the treatment of a growing number of infectious diseases. As a result, there is an urgent need for developing new drugs which are effective against current antibiotic resistant pathogens. Actino-mycetes have been proven as a potential source of bioactive compounds and the richest source of secondary metabolites (22). The isolation of antibacterial compounds from the freshwater environment is of interest to isolate novel bioactive actinomycetes. Actinomycetes form 10% of the total bacteria colonizing marine aggregates. Marine habitat has been proven as an outstanding and fascinating resource for innovating new and potent bioactive producing microorganisms. Only very few reports are available on the occurrence and distribution of antagonistic actinomycetes in the marine environment. Recent investigations indicate the tremendous potential of marine actinomycetes, particularly *Streptomyces* species as a useful and sustainable source of new bioactive natural products (1). The present study was aimed to isolate actinomycetes from marine environment and screen them for the production of secondary metabolites. The medium was supplemented with nystatin to eliminate the fungal contamination. The same method was previously done by Sambamurthy and Ellaiah (1974) that used amphotericin B as antifungal agent (23). The production of antibiotic substance is dependent on sea water (24). In this study also, the SCA, ISP 2 and Kuster's agar were prepared using sterile sea water. Okazaki and Okami (1972) observed that *Streptomyces* species showed efficient antagonistic activity (25). Holt *et al*. (1994) identified the isolated actinomycetes based on the colony morphology and gram staining (26). We have identified the actinomycetes by the presence of powdered colonies on the surface of agar plate. Actinomycetes are gram positive and filamentous in nature. Kokare* et al.* (2004) have stated the filamentous nature of actinomycetes which are gram positive (27). During the screening of the novel secondary metabolites, isolated actinomycetes showing more activity against gram positive bacteria than gram negative bacteria were often encountered. This was similar to the findings of another study (27). It has been found that estuarine actinomycetes, which remained largely ignored, show promising antibacterial activities (28, 29). In the present study, 44 actinomycetes were isolated from sediments and 24 showed wide range of inhibition zone in the primary screening. More yield of crude extract was produced with ethyl acetate solvents. The reason for the increased yield was due to the lack of water and complete miscibility in organic solvents (ethyl acetate) of the growth supernatant. Thus, the results of this investigation revealed that the marine actinomy-cetes collected from the sediments of Caspian Sea might be a potent source of novel antibiotics. It is anticipated that isolation, characterization and study of actinomycetes can be useful for the discovery of novel species of bacteria producing bioactive compounds.
